# Orchestrating pH levels in plants

**DOI:** 10.7554/eLife.91025

**Published:** 2023-08-30

**Authors:** Elke Barbez

**Affiliations:** 1 Center for Integrative Biological Signalling Studies (CIBSS), University of Freiburg Freiburg Germany; 2 Institute of Biology II, Division of Molecular Plant Physiology (MoPP), University of Freiburg Freiburg Germany

**Keywords:** auxin, root, pH, *A. thaliana*

## Abstract

The growth of a plant root relies on careful control of root surface pH.

**Related research article** Serre NBC, Wernerová D, Vittal P, Dubey SM, Medvecká E, Jelínková A, Petrášek J, Grossmann G, Fendrych M. 2023. The AUX1-AFB1-CNGC14 module establishes a longitudinal root surface pH profile. *eLife* **12**:e85193. doi: 10.7554/eLife.85193.

Cell elongation is a crucial process in the growth of plants, both above and below ground. An old theory from the 1970s postulates that plant cells can stretch when the pH of the apoplast, the space outside of the cell membrane, is low (Rayle and Cleland, 1970). A plant hormone, called auxin, mediates this process by activating proton pumps in the cell membrane, leading to an increase in the number of protons into the apoplast and thus a lower pH. This in turn, activates specific enzymes that help to loosen the cell wall and thus cell expansion (Rayle and Cleland, 1970).

In the following decades, scientists remained intrigued to unravel the molecular mechanism behind this ‘acid growth theory’. Now, in eLife, Matyas Fendrych and colleagues – including Nelson Serre and Daša Wernerová as joint first authors – report new insights into the role of pH in the growth of plant roots in the model organism *Arabidopsis thaliana* (Serre et al., 2023).

Serre et al. used a pH-sensitive fluorescent dye in combination with vertical spinning disk microscopy to visualize and quantify the surface pH of the root organs. This revealed a distinct pattern of pH variation on the root surface: acidic domains in the meristem (where cells divide) and the differentiation zone (where cells mature), and a pronounced alkaline domain in the transition zone (where cells stop dividing and prepare for elongation; Figure 1).

**Figure 1.**
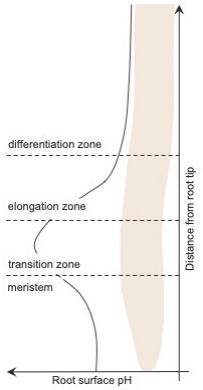
Variation in the plant root surface pH. In *A. thaliana,* the pH of the surface of the root (black line) varies with distance from the tip of the root. In the meristem – the region nearest the tip – the surface pH is acidic, but it increases to become alkalic in the transition zone, before falling again to become more acidic in most of the elongation zone and in all of the differentiation zone.

Moreover, the researchers – who are based at Charles University in Prague, the Czech Academy of Science, and the Heinrich-Heine-University in Düsseldorf – were able to demonstrate that the cellular auxin import is essential for the formation of the alkalic root surface area in the transition zone. The plant hormone auxin is necessary for plant growth and development and its optimal functionality depends on its precise distribution across plant organs and tissues (Vanneste and Friml, 2009). Serre et al. found that mutants lacking the cellular auxin importer AUX1 were unable to establish an alkalic surface domain.

Previous research has shown that once inside the cytoplasm, auxin binds to specific receptors and triggers either a slow response (which involves changes in the expression of downstream genes) or a fast response which does not require altered gene transcription (Dubey et al., 2021). Serre et al. revealed that the correct establishment of the root surface pH pattern requires the fast auxin response machinery involving the auxin receptor AFB1.

The regulation of membrane-based proton pumps is central in the acid growth theory. Serre et al. show, however, that the auxin-induced alkaline root zonation was not caused by altered proton pump activity, suggesting a distinct mode of action. Interestingly, one of the known fast auxin responses involves an intracellular increase in calcium signaling (Shih et al., 2015). Such a signaling increase has been shown to go hand in hand with pH regulation in cells (Monshausen et al., 2011; Behera et al., 2018). In particular, the calcium channel CNGC14, which enables calcium import as a fast response to auxin, has an important role in helping roots to grow along the gravity vector (Fendrych et al., 2018; Shih et al., 2015). Serre et al. found that CNGC14 was also important to establish the alkalic surface domain in the transition zone.

A rapid auxin response and its link to calcium signalling may enable the roots to adapt swiftly to auxin fluctuations and environmental cues. Indeed, Serre et al. showed that mutant seedlings with altered pH zones were less able to navigate their roots in response to external cues.

The work of Serre et al. provides new insights into the mystery of pH-driven root growth and opens further fascinating questions. While the pH in the apoplast of root epidermal cells is acidified in the transition zone, the surface pH above is alkaline (Barbez et al., 2017; Moreau et al., 2022; Monshausen et al., 2011; Serre et al., 2023). This suggests that plants can spatially differentiate the pH within and at the surface of their root organ. A cell type-specific assessment of the molecular players, such as AUX1, AFB1 and CNGC14, will provide more insight into how these variations are jointly orchestrated. It will also be interesting to see if and how environmental factors, such as external pH fluctuations or other abiotic stresses, affect the root surface pH landscape.

In summary, Serre et al. illustrate that the root surface of *A. thaliana* features distinct pH zones, particularly highlighting the alkaline region in the transition zone. By identifying three molecular players in this process – AUX1, AFB1 and CNGC14 – they provide further molecular evidence to support the role of fast auxin responses in the pH-dependent regulation of root growth.
